# Modified Ion Source for the Improved Collisional Activation of Protein Complexes

**DOI:** 10.1021/jasms.3c00071

**Published:** 2023-03-31

**Authors:** Robert L. Schrader, Thomas E. Walker, David H. Russell

**Affiliations:** Department of Chemistry, Texas A&M University, College Station, Texas 77843, United States

**Keywords:** quadrupole mass spectrometry, native mass spectrometry, Orbitrap mass spectrometry, GroEL, ion trap

## Abstract

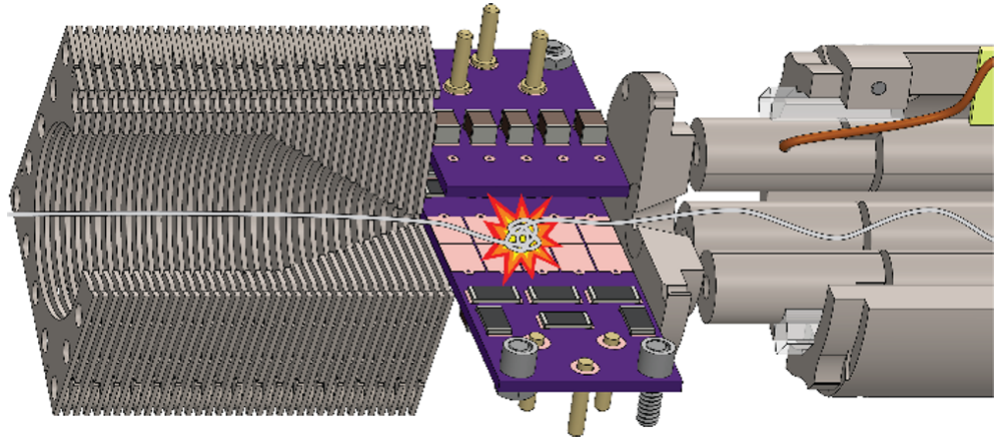

The analysis of large molecules is challenging, as they often have salts and adducts retained through the electrospray process, which increase the observed mass and compromise the achievable mass resolution. Mild collisional activation has been shown to be very effective for the removal of adducts and increases both measurement accuracy and mass resolution of large (>100 kDa) protein complexes. Collisionally activated protein ions are more completely desolvated due to the increased number of collisions when trapped following activation. A short square quadrupole maintained at 300 mTorr by a mechanical pump was added between the ion funnel and transmission quadrupole. This configuration and operation effectively removed adducts from the 800 kDa tetradecamer GroEL as well as fragmented smaller protein complexes like C-reactive protein. Due to the gas high pressure, ions of low size-to-charge ratio, such as those in charge reducing buffers, had low ejection efficiency. We show that segmenting the quadrupole rods greatly improves signal intensity for charge reduced GroEL D398A mutant compared to nonsegmented rods when operating at high pressure.

## Introduction

Native mass spectrometry is now recognized as an important technique in structural biology, as protein complexes electrosprayed from buffered solutions retain important elements of their solution structure as gas phase ions.^1−3^ One of the major hurdles in native mass spectrometry is the removal of salts and adducts that remain on the complex in the gas phase. These adducts not only reduce the accuracy of the mass measurement but also reduce the achievable resolution.^4,5^

For native MS experiments, source conditions are important in determining the quality of the obtained mass spectra. Collisional activation is often necessary to remove retained adducts,^6^ though in some highly purified samples, high resolution is achieved without collisional activation. For membrane protein complexes, collisional activation is necessary to even observe the protein complex out of the detergent micelle.^7,8^ Optimization of the pressures in the source region of quadrupole time-of-flight (Q-TOF) instruments can be used to increase the amount of activation in the source region necessary for analysis of protein complexes.^4,9−11^ To achieve high resolution for protein complexes in Orbitrap systems, ions are trapped in the higher energy collisional dissociation (HCD) cell as opposed to the C-trap, where the increased pressure improves desolvation.^12,13^ In addition to trapping in the HCD cell, ions can also be trapped in the source region so that the desolvation occurs prior to the quadrupole mass filter.^14,15^

In this work, a previously described dual quadrupole instrument^5,16^ has been modified to include a 25 mm square quadrupole (q0), which is maintained at 300 mTorr by a mechanical pump, positioned between the ion funnel and the first quadrupole. Ion activation was achieved by a potential drop between the ion funnel and the q0 rod DC potential. Trapping the ions following activation dampens their kinetic energy prior to injection into further vacuum regions.

## Experimental Section

### Chemicals

Human recombinant C-reactive protein (CRP, solution in tris buffered saline, 2 mM CaCl_2_, 0.05% NaN_3_, pH 7.5) was purchased from Lee Biosolutions (Maryland Heights, MO). HPLC-grade water was purchased from Millipore-Sigma. Ammonium acetate was purchased from Sigma-Aldrich (St. Louis, MO). GroEL D398A was expressed and purified as previously described.^17^ CRP and GroEL were buffer-exchanged into aqueous 200 mM ammonium acetate or 160 mM ammonium acetate/40 mM triethylammonium acetate using a Bio-Rad (Hercules, CA) Micro Bio-Spin P6 Column. Solutions were adjusted to working concentrations of 1–5 μM.

A Sutter Instruments (Navajo, CA) P100 tip puller was used to pull 1.5 cm o.d., 0.86 cm i.d. borosilicate capillaries (Sutter Instruments) to a 1–5 μm tip. Protein samples were loaded into the pulled capillary, and high voltage (1.1–1.5 kV) was applied to the solution through a platinum wire.

### Instrumentation

We previously described a dual quadrupole instrument in which a voltage bias between the ion funnel and the first quadrupole was used to fragment or remove adducted species from protein complexes by collisional activation.^5,16^ The full instrument diagram is shown in Figure S1. The instrument has been modified to add an approximately 25 mm quadrupole with an *r*_0_ of 3.5 mm (q0, 0.3 Torr, 1.3 MHz, 300–500 V_pp_) between the RF ion funnel (1.5 Torr, 470 kHz, 250 V_p-p_) and the first quadrupole (Thermo Fisher, Part Number 90100–60109, 1.4 MHz, 250–900 V_p-p_). The q0 quadrupole was initially constructed from square metal rods in a polyetheretherketone (PEEK) mount. This quadrupole was replaced with a printed circuit board quadrupole with segmented rods (Figure S2) fabricated by OSH Park (Lake Oswego, OR). The first quadrupole vacuum region is maintained at approximately 8 × 10^–4^ Torr and includes a 4.75 mm *r*_0_ octopole (750 kHz, 200 V_p-p_). The next vacuum region (maintained at 2 × 10^–5^ Torr) contains an identical Thermo Fisher quadrupole operated using digital waveforms and a 3.5 mm *r*_0_ octopole (850 kHz, 200 V_p-p_). Following a skimmer, the ions enter a fifth vacuum region (maintained at 10^–5^ Torr) with a 4.75 mm *r*_0_ octopole (900 kHz, 200 V_p-p_) and enter the HCD cell of a Thermo Fisher Exactive Plus EMR Orbitrap mass spectrometer (Bremen, Germany) as previously described.^18^ DC and RF voltages are applied using a Modular Intelligent Power Supply system (GAA Custom Engineering, Kennewick, WA). Digital waveforms were applied to the second quadrupole using a custom FPGA-based low voltage waveform generator^16^ with the high voltage waveform (150 V_0-p_) applied using two DEI PXI-4140 pulsers (Directed Energy, Inc., Fort Collins, CO).

## Results and Discussion

**Figure 1:**
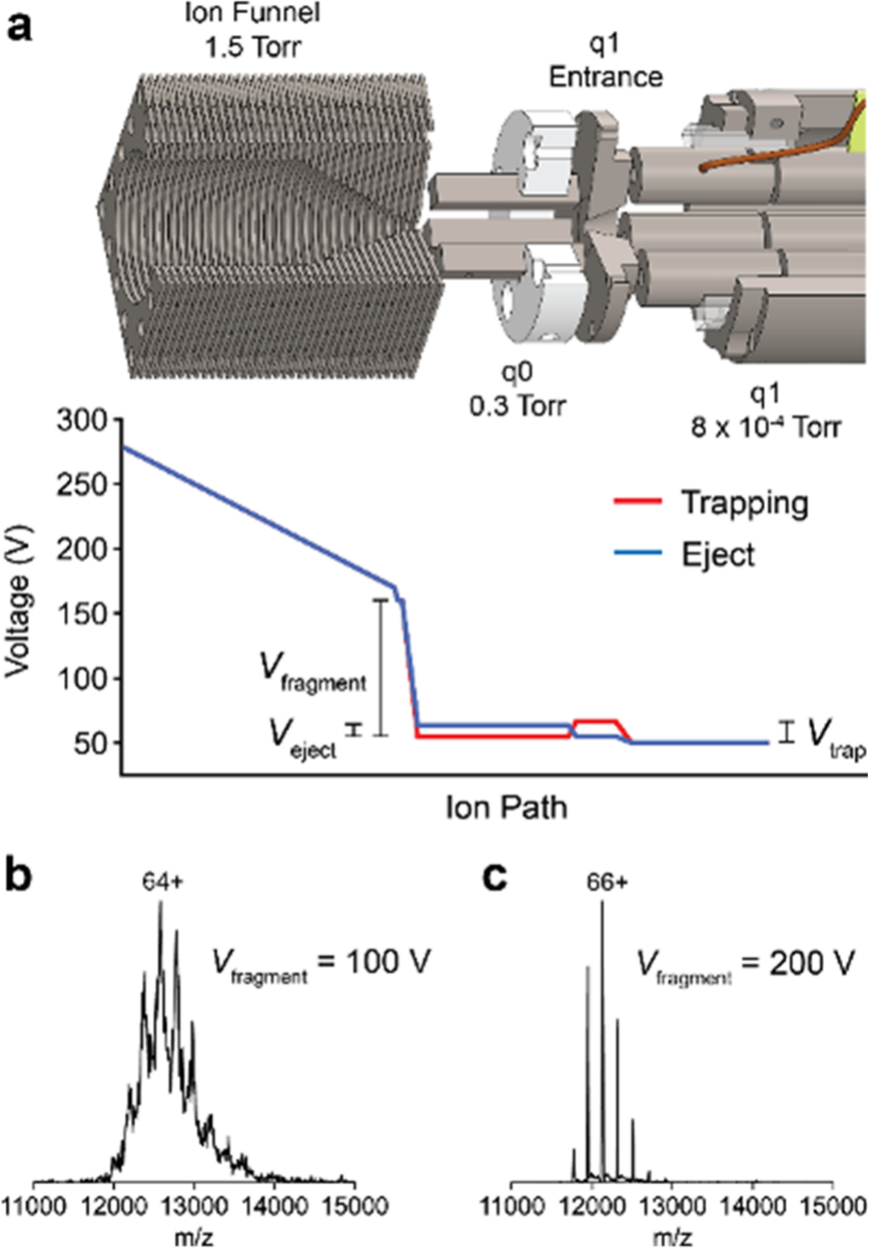
(a) A schematic of the source ion optics of the instrument is shown with pressures for each region. The voltage difference between the ion funnel and the q0 rods is referred to as *V*_fragment_, and the voltage difference between the q0 rods and the entrance of q1 during the trapping period is referred to as *V*_trap_. During the ejection period, this voltage difference is referred to as *V*_eject_. The magnitude of *V*_trap_ is small relative to *V*_fragment_ because the many collisions dampen the ion kinetic energy due to the pressure of the q0 region. GroEL spectrum acquired with *V*_fragment_ of (b) 100 V and (c) 200 V. Increased *V*_fragment_ increases the collisional activation and removes retained adducts from the complex and greatly improves the observed resolution.

To decrease the gas load on the q1 vacuum region, an additional differentially pumped region, pumped by an additional mechanical pump, containing a 3.5 mm *r*_0_ square quadrupole was added between the ion funnel and q1 (Figure 1). This reduced the pressure in q1 from approximately 6 to approximately 0.8 mTorr. In previous instrument configurations, the ion funnel, maintained at 1.5 Torr, directly interfaced with the first quadrupole region.

To achieve collisional activation in q0, the ions were accelerated by a voltage drop between the ion funnel exit and q0 rod DC voltage (*V*_fragment_). To trap the ions, the q1 entrance lens voltage was raised 1–10 V above the q0 rod DC voltage (*V*_trap_). The value of *V*_trap_ was determined by constantly applying the trapping potentials and determining the voltage necessary to stop transmission of all ions. The voltage necessary to trap the ions is low due to the high pressure in q0 (300 mTorr) where the ions undergo many collisions to dampen the kinetic energy gained from *V*_fragment_. Following the trapping period of 4 ms, the q0 rod DC voltage was raised, and the q1 entrance lens voltage was lowered (*V*_eject_) to eject the ions from the trap and into q1. The trapping scheme implemented here is similar in operation to “In-Source Trapping” implemented on the Thermo Fisher Q Exactive Orbitrap UHMR.^12^

The performance of the q0 trap was evaluated for the large protein complex GroEL. A mass spectrum of the D398A mutant of GroEL was collected with a *V*_fragment_ of 100 V (Figure 1b). In this spectrum, the charge states are barely separable from the noise, and the deconvoluted mass is 805.2 kDa, much larger than the theoretical mass of 800.4 kDa. The additional mass is attributed to the presence of salts and adducted species retained by the complex. By increasing *V*_fragment_ to 200 V (Figure 1c), the resolution is greatly increased, and baseline resolution is achieved for the charge state distribution, which is attributed to the collisional activation removing the adducted species as the deconvoluted mass is now 800.4 kDa. No fragment ions of GroEL were observed at a *V*_fragment_ of 200 V, and beyond this voltage, no signal was observed. We hypothesize that this voltage does not fragment GroEL due to the high pressure in q0.

**Figure 2:**
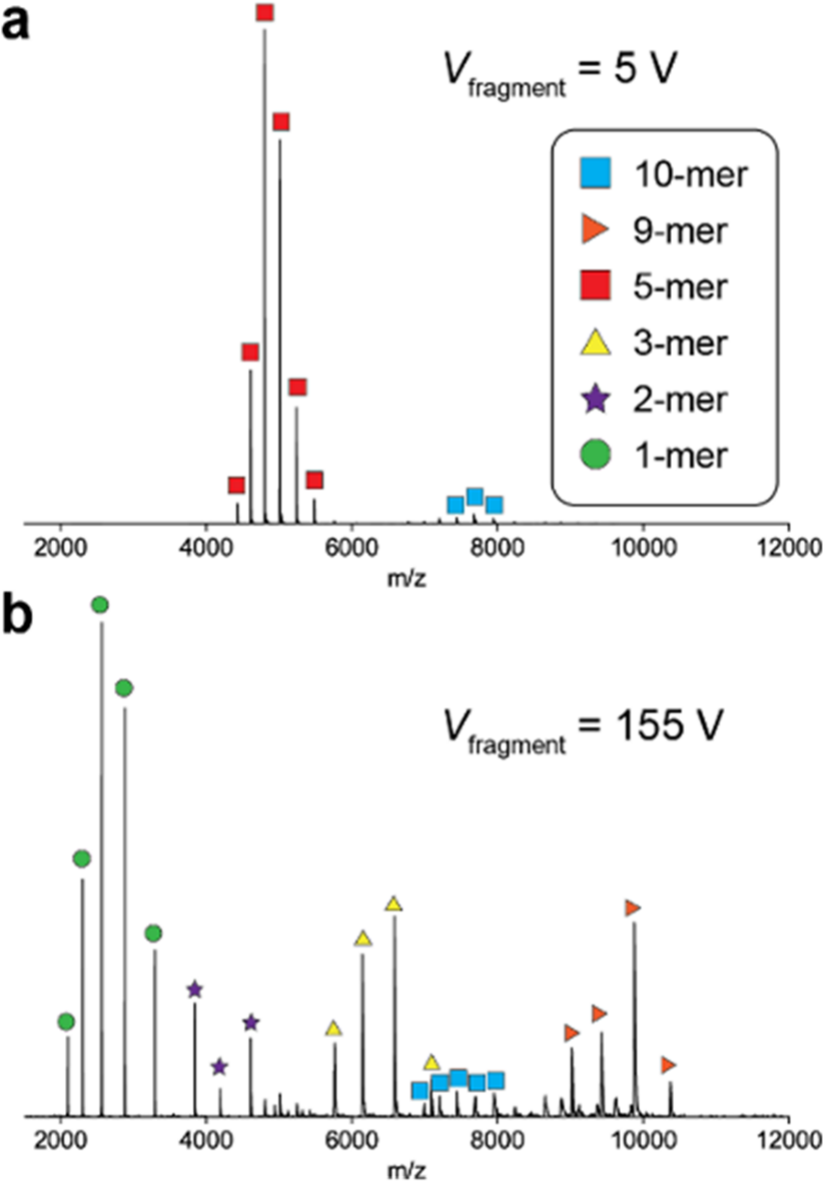
Activation of CRP in q0 using a *V*_fragment_ of (a) 5 V and (b) 155 V. Increasing voltage breaks apart both the tetramer complex and the decamer complex to give diagnostic fragmentation patterns.

In addition to removal of adducts demonstrated for GroEL, protein complexes can also be fragmented to give diagnostic fragment ions. An example of this is shown for CRP (Figure 2). For larger values of *V*_fragment_, the pentamer complex begins to form fragment monomers, dimers, and trimers, and the decamer complex begins to form fragment nonamers. These fragments span a large *m*/*z* range from approximately *m*/*z* 1000 to 10 000, showing that a large mass range can be both trapped and ejected. As these fragments are generated prior to Q2, these fragments can be mass selected and subjected to further fragmentation in the HCD cell (pseudo-MS^3^).

**Figure 3:**
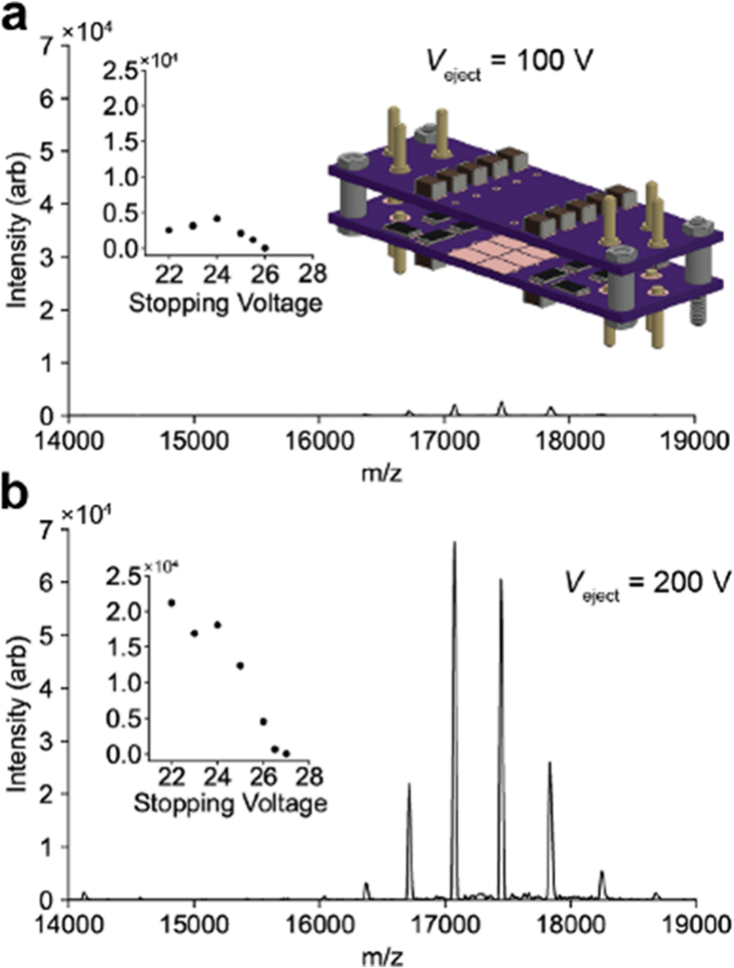
Charge reduced GroEL with *V*_fragment_ = 200 V, and *V*_eject_ of (a) 100 V and (b) 200 V shows that high ejection voltages are necessary for the efficient ejection of charge reduced proteins. Stopping curves for each *V*_eject_ show that the ion energy is unaffected by the large ejection voltage due to the high pressure in the q0 region collisionally cooling the ions.

After the ions are cooled to the center of the trap during the trapping period, it is necessary to reaccelerate them to exit the q0 region. When applying the ejection potentials, the DC field in the center of the multipole is low relative to the ends of the multipole.^19^ Multiple methods have been developed to increase the DC field within a multipole.^19−24^ Poor ejection efficiency was most pronounced for ions of low charge-to-size ratio. These ions still experience many collisions, but electric force is greatly reduced due to the low number of charges. To improve ejection efficiency, the square metal quadrupole was replaced with a planar quadrupole geometry shown in Figure 3. The source ion optics and voltage parameters with this geometry are shown in Figure S2. In this geometry, the rod pairs were divided into five segments connected via a resistive divider. During the injection period, the DC potential across the multipole was held constant. During the ejection period, the first DC segment was increased by *V*_eject_, and the final DC segment remained at the injection potential. This makes the potential across the multipole *V*_eject_ during the ejection period. SIMION simulations (Figure S3) show that ions are unable to be ejected from the square metal q0 but are readily ejected from the segmented PCB q0. As this simulation uses a hard-sphere collision model and neglects the effect of gas flow, this demonstrates the importance of gas flow on the ejection of ions from the metal q0. GroEL D398A was charge reduced by buffer-exchanging into a solution of 160 mM ammonium acetate/40 mM triethylammonium acetate. The observed ion signal for a *V*_eject_ of 200 V was increased by an order of magnitude versus a *V*_eject_ of 100 V (Figure 3). A stopping curve for each value of *V*_eject_ shows that the ion energy (at the exit of Q2) is unaffected by the large ejection voltage because of the collisional cooling in the q0 region.

## Conclusions

The addition of a short 25 mm square quadrupole (q0) between the ion funnel and first quadrupole region allowed for activation in the q0 region by a large potential drop into q0. Following activation, the ions were trapped in q0 to dampen their kinetic energy prior to entering the remaining vacuum regions. The collisional activation was shown to be sufficient to remove retained adducts from GroEL D398A, an 800 kDa protein complex, as well as fragment ions of C-reactive protein, a 115 kDa protein complex. Ejection efficiency was poor for ions of low charge-to-size ratio due to the high pressure (300 mTorr) in the q0 region, whereas the segmented PCB quadrupole improved the ejection efficiency for charge reduced GroEL D398A by an order of magnitude.

## Supporting Information Available

Instrument diagram; schematic of source optics with segmented PCB q0; and SIMION simulations comparing both q0 geometries (PDF)
