# Supplementary material for: Modified Ion Source for the Improved Collisional Activation of Protein Complexes
Source: J Am Soc Mass Spectrom. 2023 Mar 31;34(5):977–80. doi: 10.1021/jasms.3c00071 (PMC10510017; doi:10.1021/jasms.3c00071)
Supplement: Supplementary file 1 — js3c00071_si_001.pdf [file js3c00071_si_001.pdf]

**Supplementary Materials**

# Modified Ion Source for the Improved Collisional Activation of Protein Complexes

Robert L. Schrader, Thomas E. Walker, and David H. Russell*

Department of Chemistry, Texas A&M University, College Station, TX 77843

*Corresponding author; email: russell@chem.tamu.edu

Table of Contents

| | Page |
|---|---|
| Figure S1 | S2 |
| Figure S2 | S3 |
| Figure S3 | S4 |

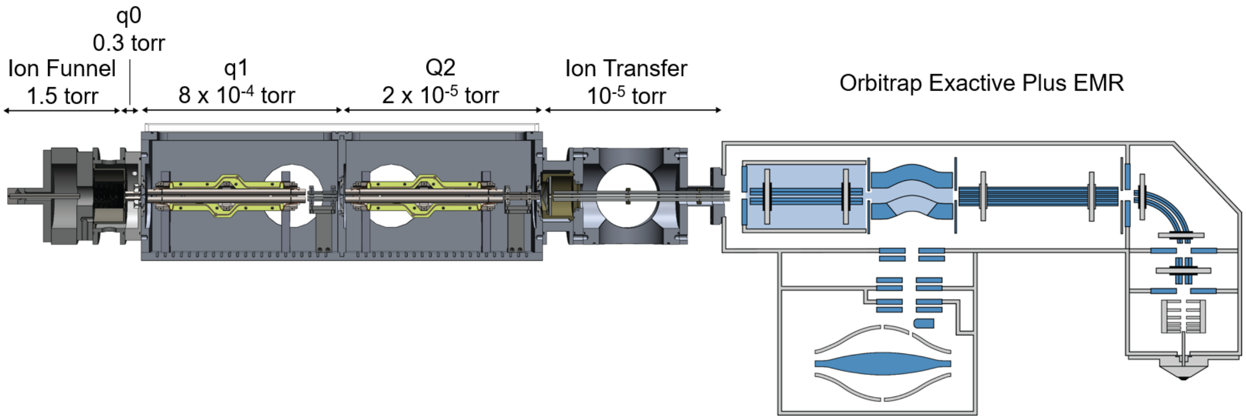


**Figure S1.** Solidworks rendering of the instrument interfaced with the rear of the HCD cell of the Orbitrap Exactive Plus EMR with major components labeled with approximate pressures of each vacuum region.

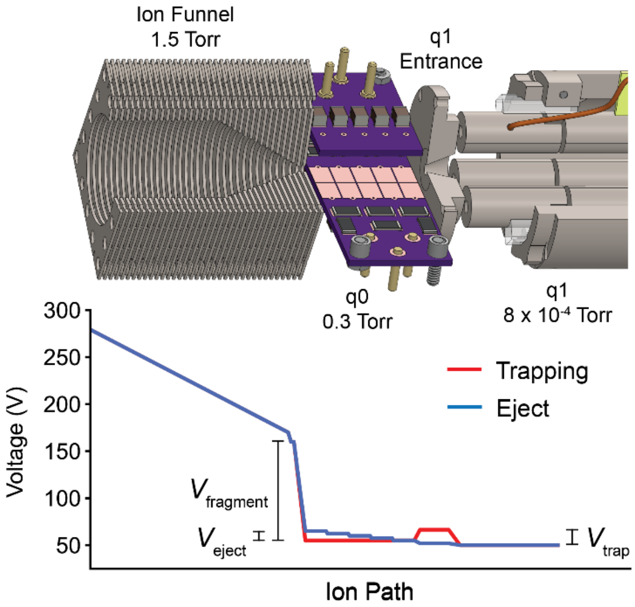


**Figure S2.** Schematic of the source optics with modified q0 to improve ejection efficiency. The RF electrodes are split into 5 equal sections separated by 0.01 in and connected by a resistive divider. During the injection period the DC is constant across each of the sections. During the ejection period the front DC is raised to create a DC gradient along the ion axis.

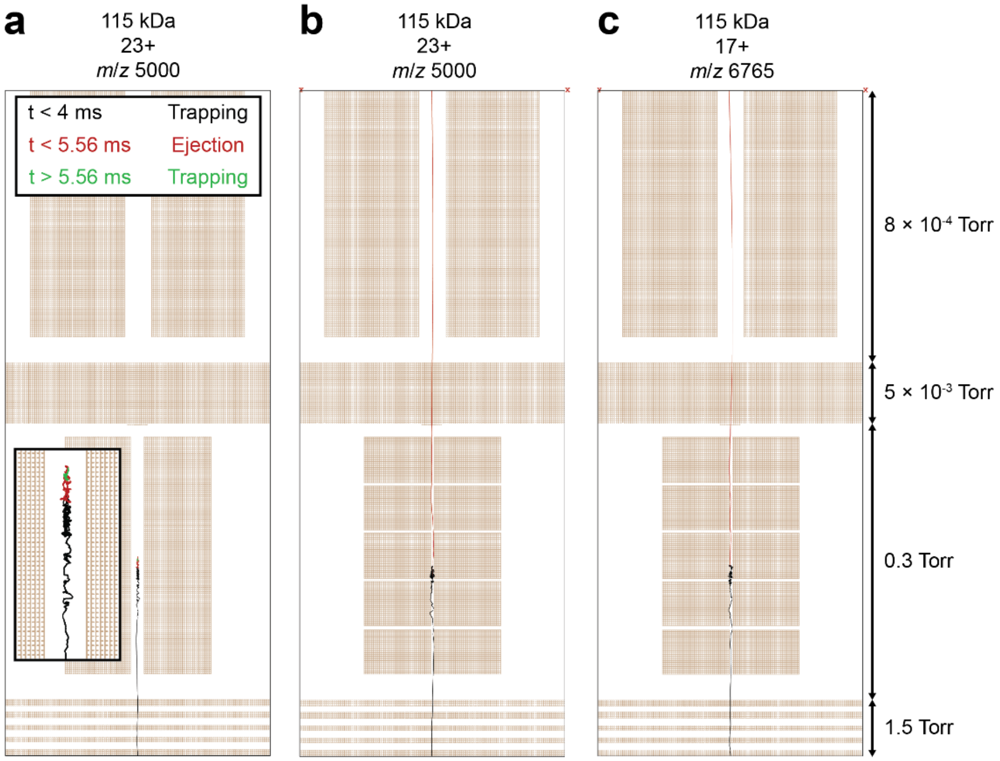


**Figure S3.** SIMION simulations of (a) metal q0 and (b,c) segmented PCB q0 for 23+ and 17+ charge states of CRP. The HS1 collision model was used, and uniform pressures were set for each region. For the metal q0 the ion is not ejected from the trap within the ejection period (red), whereas for the segmented PCB q0 both the 23+ and 17+ charge state are ejected within the ejection period.
